# Possible Use of Bacteriophages Active against *Bacillus anthracis* and Other *B. cereus* Group Members in the Face of a Bioterrorism Threat

**DOI:** 10.1155/2014/735413

**Published:** 2014-08-28

**Authors:** Ewa Jończyk-Matysiak, Marlena Kłak, Beata Weber-Dąbrowska, Jan Borysowski, Andrzej Górski

**Affiliations:** ^1^Bacteriophage Laboratory, Ludwik Hirszfeld Institute of Immunology and Experimental Therapy, Polish Academy of Sciences, Weigla 12, 53-114 Wroclaw, Poland; ^2^Phage Therapy Unit, Ludwik Hirszfeld Institute of Immunology and Experimental Therapy, Polish Academy of Sciences, Weigla 12, 53-114 Wroclaw, Poland; ^3^Department of Clinical Immunology, Transplantation Institute, The Medical University of Warsaw, Nowogrodzka 59, 02-006 Warsaw, Poland

## Abstract

Anthrax is an infectious fatal disease with epidemic potential. Nowadays, bioterrorism using *Bacillus anthracis* is a real possibility, and thus society needs an effective weapon to neutralize this threat. The pathogen may be easily transmitted to human populations. It is easy to store, transport, and disseminate and may survive for many decades. Recent data strongly support the effectiveness of bacteriophage in treating bacterial diseases. Moreover, it is clear that bacteriophages should be considered a potential incapacitative agent against bioterrorism using bacteria belonging to *B. cereus* group, especially *B. anthracis*. Therefore, we have reviewed the possibility of using bacteriophages active against *Bacillus anthracis* and other species of the *B. cereus* group in the face of a bioterrorism threat.

## 1. Introduction

Shortly after the September 11 terrorist attacks using captured planes, letters containing anthrax spores were posted to news media and the US Senate. Five people died and several others survived the disease. This incident proved that bioterrorism using* B. anthracis* is a real danger and society needs an efficient weapon to neutralize this threat. Experts believe that if such an attack occurred in a large city, hundreds of thousands of people could be at risk of the deadly disease, while the present systems of defense are insufficient.

Anthrax is an infectious disease with high epidemic potential (characterized by high morbidity and mortality, with real possibility of being used in a bioterrorism attack with spores of* B. anthracis*). The pathogen (especially spores) that is the cause of anthrax may be transmitted in human populations by way of aerosolization (natural or artificial), resulting in epidemics with high mortality [1]. These bacteria are rare in the USA and highly infective and pose a huge threat to public health [2]. There is no strictly defined infectious dose for humans. Its amount may be influenced by such factors as the route of infection (type of anthrax), state of health of the infected person, and virulence of the infecting strain [3]. The disease caused by* B. anthracis* is treatable with antibiotics (such as penicillin G, amoxicillin, or ciprofloxacin), but the prognosis depends on the time after which the pathogen is identified and the application of appropriate therapy [4].

Anthrax spores are easy to store, transport, and disseminate and may survive in soil for many decades. Due to this feature,* Bacillus anthracis* is likely to be used as a bioterrorist weapon [5]. Moreover, the bacteria produce dangerous toxins. Antibiotic resistance in bacteria from environmental samples may cause serious problems in treating anthrax. The capsule that can be present in some strains could be dangerous as it inhibits phagocytosis of these bacteria. Moreover, the* B. cereus* group—which* B. anthracis* belongs to—consists of very homogeneous bacteria with close relatedness. This feature may pose problems with identification of and differentiation between bacteria belonging to this taxonomy group.

Bacterial viruses, bacteriophages (phages), are natural enemies of bacteria and recent data strongly suggest their effectiveness in treating bacterial diseases including those caused by antibiotic-resistant microbes [6]. Their biology and current applications have been recently summarized in detail [7]. Therefore, it is clear that phages should also be considered a potential tool against bioterrorism using* B. anthracis *or other* B. cereus* group bacteria.

## 2. *B. anthracis* as a Bioterrorism Tool

### 2.1. Pathogenesis of* B. anthracis* and Other* B. cereus* Group Bacteria

The etiological agent of anthrax,* Bacillus anthracis*, is a Gram-positive, aerobic or facultatively anaerobic, spore-forming, and rod-shaped bacterium, which appears in cell chains.* B. anthracis*,* B. anthracis*, together with four other species (*B. cereus* sensu stricto,* B. thuringiensis*,* B. mycoides*, and* B. weihenstephanensis*) constitute the* Bacillus cereus* group [8]. This zoonotic pathogen is mainly present in soil, water, and animals. It infects animals and, occasionally, humans [5, 9]. There is a risk of passive transfer of anthrax from animals to humans through insects [5]. Spores produced in the presence of oxygen [10] are stable and resistant to harsh external conditions like heat, cold, pH, desiccation, and chemicals. They can germinate when exposed to a nutrient-rich environment, such as the tissues or blood of an animal or human host [11]. The climate may directly or indirectly influence the way in which an animal comes into contact with the spores or affect the general state of a host's health and the level of their resistance to infection [5]. There are data [5] regarding the impact of various factors, such as rainfall, temperature, state of the host, and population density, on the epidemiology of anthrax; however, there is no agreement on the roles played by these factors in the incidence of the disease. Unfortunately, there are no hard scientific data to support these theories.

In humans there are three main forms of this disease, cutaneous, gastrointestinal, and inhalational, according to the route of infection [12]. Each of these forms can be lethal. The most dangerous is the inhalation form (it may be induced by 8 × 10^3^–5 × 10^4^ spores). In people untreated, death occurs in 97 to even 100% of cases within 3–5 days, but in people treated already at the early stage of the disease the mortality rate is reduced to 75%. On the other hand, mortality in untreated cutaneous form of anthrax can range from 10 to 20%, and in treated cases it is below 1% [4, 13].

The virulence of anthrax is associated with the production of poly-D-glutamic acid polysaccharide capsule (PDGA) [14, 15]. The* cap* gene encoded on the pOX_2_ plasmid is responsible for the synthesis of the capsule. The mechanism of inhibition is not well established [16]. One of the possibilities may be phagosomal escape. Moreover, it is suggested that the capsule may block bactericidal activities of neutrophil cationic peptides, for example, *α*- and *β*-defensins. It has been demonstrated that the capsule is poorly immunogenic and evades recognition as an antigen by the immune system because it protects the surface antigens and protects bacterial cells from the circulating antibodies, therefore enabling the spread of bacilli inside the host body [17]. Moreover,* B. anthracis* strains produce toxins that consist of three peptides: protective antigen (PA), lethal factor (LF), and edema factor (EF). These peptides are conditioned on the pOX_1_ plasmid where three genes,* pag*,* lef*, and* cya*, are located. The LF protein in combination with PA creates a lethal toxin, but EF with PA forms an edema toxin. Anthrax toxins are produced by vegetative forms of bacteria. In combination, virulence factors promote the multiplication of bacilli after invading the human organism. The lethal toxin causes the release of tumor necrosis factor (TNF) and interleukin-1 (which are responsible for rapid health deterioration during the inflammatory process) from macrophages, as well as development of symptoms and, possibly, cell damage [18, 19]. The edema toxin causes the formation of edema in tissue as a result of water and Cl^−^ ions loss from cells and may inhibit neutrophil phagocytic activity and oxidative burst [18]. Anthrax can also result in necrosis, septicemia, organ failure, and death. If not treated, patients may die in a few days.

Initially, the symptoms of anthrax are nonspecific (symptoms are difficult to distinguish from those of other diseases); therefore, it is difficult to recognize and quickly apply proper treatment [3]. However, at the initial stage of the disease, people should be treated with antibiotics or vaccinated as early as possible, as progression of the disease (especially in the case of inhalational anthrax) is rapid, and if the treatment is not applied within the first 24 h from first observed symptoms, it may result in death.

In some cases, the consumption of contaminated food (meat and milk) has led to foodborne illnesses associated with* B. anthracis* [20]. Conversely, the closely related species* B. cereus* is responsible for the majority of foodborne illnesses attributed to the* B. cereus* group. There is a broad range of foods associated with* B. cereus* infection including food of both animal and plant origin. Many of these foods may contain* B. cereus* since spores of this organism are heat-resistant and can survive cooking [20]. Food poisoning by* B. cereus* is a result of food-contaminating enterotoxins—emetic (vomiting) and diarrhogenic—that are produced by the bacteria. The first toxin causes intoxication as a result of thermostable toxin (cereulide, cyclic peptide toxin) ingestion, while the second, diarrheal one is an effect of infection by vegetative cells or spores producing heat-labile enterotoxin in the small intestine [21]. The symptoms of emetic poisoning occur within 1–5 h after ingesting contaminated food. This toxin is produced during bacterial growth in food [22]. Strains that are able to cause diarrhea are difficult to identify because of the diverse and complicated mechanisms characterizing this type of infection. The symptoms of diarrheal syndrome occur 8–16 h after food ingestion [23].

### 2.2. Epidemiology of* B. anthracis* and Other* B. cereus* Group Bacteria

Nowadays, the risk of anthrax is extremely small, at least in developed countries, where animal husbandry is carried out in modern conditions and hygiene is respected. Anthrax may constitute a problem especially in countries where the vaccination of animals is not practiced. However, the risk of bioterrorism using the pathogen is also a real threat.

There is no strictly defined infectious dose for humans. Its amount may be influenced by such factors as the route of infection (type of anthrax), state of health of person, and virulence of the infecting strain [3]. The infectious median lethal dose (LD50) is likely within the range of 2500–55000 spores [24]. But there are data indicating that for induction, cutaneous anthrax 10 or fewer spores are required [25]. In the case of gastrointestinal anthrax, however, the defined minimal infectious dose (Mid50) is estimated to be approximately 10^11^ spores [26]. Epidemiological evidence suggests that the majority of cases of foodborne illness caused by* B. cereus* have been associated with concentrations in excess of 10^5^ cfu/g in food. Only rare cases of illness involving 10^3^–10^5^ cfu/g of* B. cereus* in food have been reported [27]. Both* B. cereus* and* B. anthracis* bacteria may infect people, but more serious side effects may be observed in immunocompromised, young or old patients in particular [20]. For example, skin injuries may be a convenient way of anthrax spreading; for example, soil contaminations may be dangerous especially for patients predisposed to bacterial infections, such as those suffering from diabetic foot syndrome, because of nonhealing ulcers that may constitute the way for spores or vegetative forms to invade the human organism.

Natural* B. anthracis* is present in the environment; for example, the highest level of anthrax spores has been detected in Namibia, where in the vicinity of animal carcasses it amounted to 1 000 000 spores per 1 g of soil [28]. Using anthrax bacilli for bioterrorism purposes requires much higher doses [2]. Data show that 100 kilos of powdered spores may be a lethal dose for 10^13^ people.

A simulation of an expert committee of the World Health Organization [5] showed that the release of 50 kilos of anthrax spores over a city would result in 250 000 infections leading to 95 000 deaths (without treatment). The cost of a bioweapon attack using anthrax was estimated at $26.2 billion per 100 000 people exposed to the biowarfare agent [24]. Turnbull et al. indicated that the highest levels of anthrax spores (20 to 40 colony-forming units of spores per cubic meter were detected) were found in air at dusty anthrax carcass sites in Namibia, 3 to 9 m above those sites [5]. Interestingly, the results of estimation indicated that it would take about 2.5 minutes for a human to inhale 1 spore of* B. anthracis*. But the authors suggested that the probability of inhaling anthrax spores depends significantly on the size of the particles to which spores are attached [3].

The Convention on the Prohibition of the Development, Production, and Stockpiling of Bacteriological (Biological) and Toxin Weapons of 1971 prohibits conducting research using bacteria (e.g.,* B. anthracis*), their toxins, and viruses, for offensive purposes. It does, however, permit the development of vaccines for defensive purposes. There are speculations yet that somegovernments fund the conduct of research concerning* B. anthracis* application as a biowarfare agent [29]. The epidemic in the Sverdlovsk military laboratory (1979) was caused by accidentally releasing aerosol containing anthrax spores (probably 1-2 g), which were carried by the wind and which caused the greatest ever documented epidemic of pulmonary anthrax in human history. Moreover,* B. anthracis* could also be involved in cases of unintentional spread of bacteria, as recently happened when a laboratory mix-up exposed many employees to anthrax [30]. The 2012 report showed that decontamination after the anthrax letters attacks from 2001 in the US, as a result of which 11 cases of anthrax inhalation (five patients died) and 11 cases of cutaneous anthrax were reported, costed $320 million [31].

*B. anthracis* may be attractive as a biological weapon due to low production costs and ease of transmission [2]. Vegetative* B. anthracis* forms are not easily transmitted, but spores can be transmitted to humans, and therefore applying spores in the aerosol form is probably the most effective. The anthrax bacillus is easy to obtain in culture and the costs of spore production are low; it is estimated that the production of 1 kilo of spores averages $50 [2, 32]. The source of infection may be anthrax spores contained in aerosol or foods. After release, the anthrax aerosol is odorless and invisible and may be transferred over a long distance (many kilometers). Spores are robust and long-lasting (they are resistant to heat, chemicals, ionizing radiation, and ultraviolet light) [19]; for example, spores that were isolated in Kruger National Park in Africa from animal bones were estimated to survive about 200 years [33]. Boiling spores in water for 10 minutes causes their complete destruction [18]. Bacteria that belong to the* B. cereus *group are widespread and able to form spores which have the ability to remain resistant despite long-term storage and show thermostability. These are the reasons for the existence of a wide variety of foodborne illnesses.

*B. anthracis* is usually a drug-sensitive strain, but strains that may be multidrug resistant are deliberately engineered [4]. A potential* B. anthracis* terrorist attack may be caused by contamination of food and water, spread by letters, or spraying in public transport. It may cause widespread panic and requires special, quickly arranged actions for collective health preparedness. Results of anthrax attack simulation demonstrate that aerosol spores penetratethroughout a building in less than 4.5 min [34]. What is more is that prompt action, such as closing the doors and windows, shutting the ventilation system, and deactivating heating or air conditioning, would effectively reduce spore concentration inside the site in which the aerosol was released [35].

## 3. *B. anthracis* Bacteriophages

Bacteriophages (phages) are viruses that infect and multiply only in bacterial cells. It is estimated that their abundance in the biosphere exceeds 10^30-31^ virions [36, 37], ten times more than bacterial cells [38]. Bacteriophages are present in the environment: soil, marine water [39], and extreme conditions such as the Sahara desert sands or hot springs [37, 38]. We consume them with food and drinking water. Together with bacteria they constitute an integral part of the microbiome [40]. Phages in humans may be successfully used in the treatment of a wide range of infections, both local and systemic [6]. Applying phage therapy is safe for patients. There has been low incidence of phages' adverse effects (e.g., nausea, loss of appetite, superinfection, and body temperature increase) associated with the use of them [6, 41]. The results obtained by Łusiak-Szelachowska et al. (2014) indicated that the induction of antiphage antiserum activity in patients receiving phage therapy does not influence the final outcome of the therapy [42]. Despite these data, phage therapy (regarding the use of different phages or different cocktails consisting of different phages) has not been approved by the FDA so far. Clinical trials that may confirm the safety and effectiveness of the therapy need to be conducted [6].

Interestingly, bacteria's resistance to antibiotics does not contribute to the formation of phage resistance [43]. Therefore, phages may be used to treat infections caused by antibiotic-resistant bacterial strains, for example, methicillin resistant* Staphylococcus aureus* (MRSA), vancomycin-resistant* Enterococcus* (VRE), and extended-spectrum beta-lactamases producing strains (ESBL) [44–48]. Moreover, phages may be simultaneously active against bacteria resistant to many antibiotics [47, 49].

Anthrax-specific phages were first isolated in the 1950s [50]. In 1951 McCloy isolated the lysogenic W phage from an atypical* B. cereus* strain [51]. The phage was specific to all 171 isolates from* B. anthracis* but showed limited activity against* B. cereus* strains (only 2 of 54 strains) [52]. These data indicate specific activity of the phage, especially against anthrax bacilli. Then, Brown and Cherry isolated a gamma (*γ*) phage which was the lytic variant of the W phage [53]. They demonstrated that the *γ* phage is able to lyse both encapsulated and nonencapsulated* B. anthracis *strains. However Negus et al. suggested that the optimized capsule production in* B. anthracis* tested by Brown and Cherry might have been carried out incorrectly [54]. Interestingly, the phages tested by Negus et al.—*γ*, Fah, F7, and F9—were able to lyse* B. anthracis* Sterne in both capsulated and nonencapsulated form.

Phages active against* B. anthracis* (both lytic and lysogenic) are widespread in the environment and have been isolated from soil, carcasses, feces, sewage, and the intestinal tract of the earthworm* Eisenia fetida* [54–58].* B. anthracis* specific phages and their characteristics are presented in Table 1. Apart from phages listed in Table 1, there have been many more anthrax phages isolated, for example, Nk, DB, and SP50, belonging to* Myoviridae* isolated from Iowa topsoil [55]; BA39, BA21, BA28, and BA51 isolated from a sewage treatment plant (Germantown), belonging to* Myoviridae* [59]; *ф*20 lysogenic phage induced by exposure to UV light, belonging to* Siphoviridae* [60]. Interestingly, Lee et al. prepared a review in which they characterized and collected three groups of bacteriophages infecting members of* B. cereus* group, according to their genomic analysis [61].

Bacteriophages, as well as lysins (encoded in phage genomes), could be useful in the treatment of infections caused by* B. anthracis*, destruction of* B. anthracis* germinated spores, and environmental disinfection. Treatments with phages or lysins may be extremely important because of being potentially safe for humans infected with anthrax and threatened with death caused by those bacilli. Bacteriophage-based methods for identification and/or treatment of anthrax may be methods of the future. This common and well-investigated tool—which bacteriophages constitute—is also very useful in molecular biology. There are methods of* B. anthracis* strain identification, for example, the *γ* test approved by the FDA in 2005 [1] or the bioluminescence test based on light detection after the application of a phage with the lux AB gene. Possible phenotypic alterations of temperate phages in* B. anthracis* include an influence on bacterial sporulation (the prophage state may induce rapid sporulation phenotype), biofilm formation, and induction of exopolysaccharide production [69]. However, bacteriophages may be used not only as antiterrorism tools, but also aspotential bioterrorism agents [70]. For example, lysogenic bacteriophages that contain virulence or drug resistance genes may be used for genetic manipulation, enabling the modification of nonpathogenic bacteria into a strain that would be resistant to available antimicrobial drugs. Despite the intensive studies on isolation and characterization of* B. anthracis* phages, many questions still remain unanswered.

**Figure 1:**
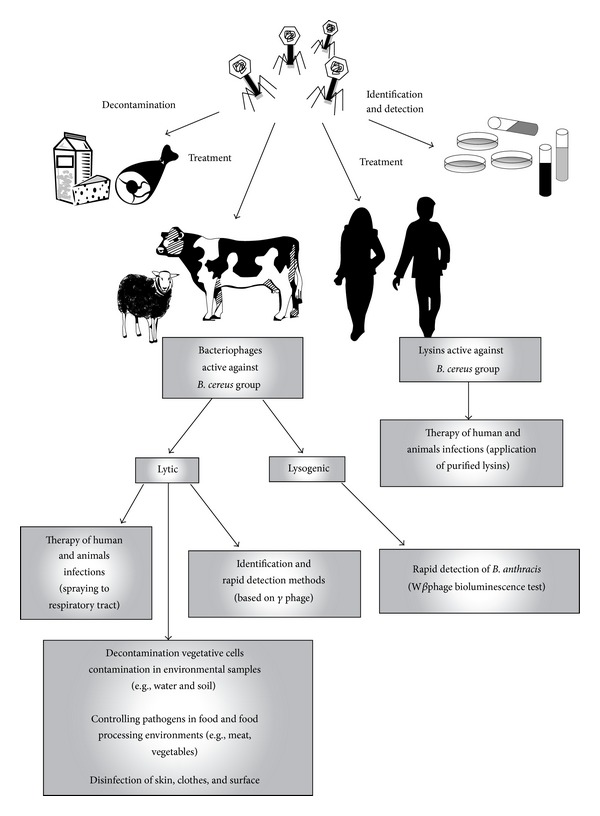
The possibility of using bacteriophages and lysins against bacteria from* Bacillus cereus* group.

Apart from previously described possible phage applications, bacteriophages can be also used in controlling bacterial pathogens from the* B. cereus* group in food and food processing environments. A summary of the potential use of phages active against bacteria from the* Bacillus cereus* group, in the case of potential use of these bacteria as a biological weapon, is presented in Figure 1.

One of the advantages of bacteriophages specific for* B. anthracis* is their narrow activity against the bacterial host, being restricted to strains of* B. anthracis*—not active against closely related strains of the* Bacillus* genus (such as* B. cereus* or* B. thuringiensis*). But bacteria from the* B. cereus* group are very closely related.* Bacillus cereus* and* B. anthracis* share many common phage parasites [71]. Close relatedness between* B. anthracis* and bacteria from the* B. cereus* group enables certain phages active against* B. anthracis* to show activity also against* B. cereus* and vice versa [72].

In our opinion, when the usefulness of anthrax phages is considered a tool for therapy and the detection of bacteria, the specificity (narrow host range) is an advantage. If the aim is decontamination, the broad lytic spectrum may be helpful in case of elimination of both* B. anthracis* and other species belonging to the* B. cereus* group is the objective.

## 4. Identification of* B. anthracis*

Should a bioterrorism attack occur, there must be a possibility for its rapid detection and identification in an average microbiological laboratory. Such work requires the biological safety level 3 use (BSL-3) [28]. To work with agents that may cause serious or potentially lethal diseases (e.g.,* B. anthracis*), especially through the inhalation route of exposure, the laboratory should be designed to ensure the personnel safety. It has to, for example, be self-closing and entered through an airlock or anteroom and have double-door access and a hand washing sink near the laboratory exit; the air cannot be recirculated, and there must be negative airflow into the laboratory [73].

*B. anthracis* may be isolated from different animal body specimens (blood cultures, cerebrospinal fluid, stool, respiratory specimens, and cutaneous lesions), but their source depends on the type of anthrax [10].

Methods used for identifying* B. anthracis* should offer rapid detection even at low concentration of the pathogen, with no crossreactivity. They should be simple to perform and possible to perform at the site of sampling [19]. Moreover, the assay should enable the detection of both spores and vegetative forms. Therefore, the real challenge is to optimize the* B. anthracis* foolproof detection system for protecting public health.

Van Tongeren et al. studied the microbial community of the interior human environment of the International Space Station [74]. They isolated from one microbial sample multiple strains belonging to the* B. cereus* group, including* B. anthracis*. The authors emphasized that there is a real challenge in rapid detection of anthrax bacilli from that type of microbiological material.

Differentiating* B. anthracis* from other strains of the* Bacillus* genus may cause diagnostic difficulties. Standard microbiological methods used in laboratories take 24–96 h [5, 62]. Despite the introduction of the Ground Anthrax Bacillus Refined Isolation (GABRI) method to analyze environmental samples, which enables the detection of low levels of* B. anthracis* [75], this method also requires 24 or 48 h of incubation. Public security requires shorter times of detection of anthrax contamination. Methods depending on antigen detection allow the result to be obtained within several hours [19] but may show a lack of sensitivity and specificity as well as crossreaction with other strains of the* Bacillus* genus, thus giving false positive results. The nucleic acid based method (e.g., polymerase chain reaction) is highly specific but does not discriminate between live and dead bacteria [76] and clean starting samples are required [19]. So far, there is no method that can be considered reliable.

### 4.1. Detection of* B. anthracis*

Phages may have potential to be used in* B. anthracis* detection in clinical, environmental, or food samples [70]. Most phage-based assays exploit the *γ* phage [77]. They are based on high specificity of phages to certain bacteria species, and their detection limit is 10^3^–10^5^ cfu/mL. It is possible to identify anthrax bacilli as quickly as within 60–120 min.

The phage *γ* test is a standard method for identification of* B. anthracis* strains and differentiates them from other closely related strains from the* Bacillus* genus [78]. This routine identification test takes 2–4 days [5]. The presence of a polypeptide capsule inhibiting* B. anthracis* infection by lytic *γ* phage constitutes a serious problem in using this method. The synthesis of the capsule blocks the GamR receptor on the bacterial cell surface responsible for phage binding [79].

Based on the differences between phages' lytic spectrum, it is supposed that the identification/typing test using the *γ* phage is probably less sensitive when compared with the Wip1 phage [58, 80]. Additionally, Wip1 plaques can be detected after merely 12 h after bacterial infection.

Detection of* B. anthracis* by using W*β* phage bioluminescence enables the detection of a signal as soon as after 16 min from the moment of commencing the infection of* B. anthracis *cells with the W*β* phage possessing an incorporated luxAB reporter gene [62]. The method allows for direct detection of* B. anthracis* in clinical specimens (e.g., blood, stool, and sputum) [77] and excludes the detection of members belonging to the* B. cereus* group closely related to* B. anthracis* [4, 62]. It detects only live bacteria (~10^3^ cfu/mL within 60 min) and* B. anthracis* germinated spores (within 60 min). Spores are refractory to phage because they do not show the GamR on their surface; therefore spores may be detected only in the germinating state. A higher phage titer gives a stronger detection signal. The limitations of the bioluminescent W*β* phage-based method may result from* B. anthracis*' resistance to phage infection and no possibility of the reporter phage to infect encapsulated strains. Abshire et al. found that only 2 strains out of 51 tested isolates of* B. anthracis* were resistant to the lysis caused by the *γ* phage [65]. It may indicate that natural phage resistance of* B. anthracis* strains is not common.

Kan et al. identified the ligand on the Wip1 bacteriophage that is highly specific to the receptor on* B. anthracis* [80]. They observed that the gene product p23 of the Wip1 bacteriophage is a receptor-binding protein on the phage surface. The presence of this protein and narrow host range of the Wip1 phage may provide new tools for the identification of* B. anthracis* strains.

The anthrax spores should be detected before the occurrence of symptoms, especially by the use of continuous monitoring of spore content in the air [81]. The system should be sensitive and selective to avoid false alarms of bioterrorist attack. Brigati et al. proposed a method based on landscape pIII phage-display libraries (that contain thousands of copies of peptides best binding to a specific antigen) and phages expressing a specific peptide used as a probe that specifically binds to* B. anthracis* spores [81]. This method is not ideal due to the possibility of clones crossreacting to other species belonging to the* Bacillus* genus. But the most specific phage display spore binding peptide EPRLSPHS bound 3.5- to 70-fold more strongly to the* B. anthracis* Sterne spores than to other strains. Also, sensors that use filamentous phages may be useful in* B. anthracis* spore identification, and wireless magnetostrictive sensors showed binding affinity to* B. anthracis* that was better than to* B. cereus* and to* B. subtilis* spores [82]. Applying filamentous phages in these methods is justified for these phages are suspected to be the most stable nucleoproteins in nature. They are extremely resistant to high temperature (even up to 80°C), acids and alkaline solutions, organic solvents (50% alcohol), and denaturing agents (6–8 mg/L urea) [81, 83]. The detection limit of the described method is at 10^3^ spores/mL [84]. Detection of anthrax spores in water using phage as a bioprobe and magnetostrictive mili/microcantilevers (MSMC) designed as a sensor platform was developed by Fu et al. [85]. This method enabled* in situ* detection. Schuch et al. prepared a rapid and highly specific system for detecting spores [86]. The detection is based on light emission in the presence of luciferin and luciferase and the release of ATP from lysed bacterial cells. It is based on the ability of PlyG to kill germinating spores and is applied using a hand-held luminometer. The signal was detected only 10 min after the addition of germinating spores of the RSVF1 strain. What is more is that the light was emitted only 5 min after adding PlyG. Moreover, a method based on the binding of* B. anthracis* vegetative cells has also been developed [87].

Shabani et al. presented a phage-modified electrode microarray method for rapid and direct impedimetric detection of* B. anthracis* [88]. It is based on the immobilization of the *γ* phage and its high specificity to* B. anthracis* species and provides a low-cost platform for direct identification of* B. anthracis*. Its detection limit is 10^3^ cfu/mL with a sample volume of merely 40 *μ*L.

## 5. Treatment of Anthrax

Without immediate treatment, inhalation of anthrax spores is usually lethal (within the first 24 h from observed symptoms, it may result in death). Therefore, therapeutic intervention should be initiated as early as possible [89]. The antimicrobial chemotherapy recommended for the treatment of patients with inhalational anthrax is effective, but long-term therapy may cause antibiotic resistance in* B. anthracis* [90]. Drugs used for postexposure prophylaxis are penicillin G, amoxicillin, doxycycline, ciprofloxacin, and ofloxacin administered for 60 days or more [24].

Penicillin has been considered the drug of choice, and it is very rare that resistance to this antibiotic is found in naturally occurring strains [9]. Ciprofloxacin, penicillin, and doxycycline are recommended for the treatment of humans and as prophylactics after exposure to the spores [91]. Many* in vitro* studies show that* B. anthracis* is susceptible to penicillins, fluoroquinolones, tetracycline, chloramphenicol, aminoglycosides, macrolides, imipenem/meropenem, rifampicin, and vancomycin [9, 91, 92]. However, the organism is resistant to cephalosporins, trimethoprim, and sulphonamides.* B. anthracis* is usually sensitive to a broad range of antibiotics. Cavallo et al. tested its sensitivity to antibiotics in 96 strains of* B. anthracis* isolated from humans (1), animals (28), and the environment (67) in France [89]. 11.5% of strains were resistant to penicillin G and amoxicillin. All of them were resistant to cotrimoxazole but susceptible to antibiotics such as doxycycline, vancomycin, clindamycin, rifampicin, imipenem, or teicoplanin.As a result of long-term antibiotic treatment* B. anthracis* strains may be converted into antibiotic resistant strains [90]. It was observed that only 11% of natural/environmentally isolated strains of* B. anthracis* were resistant to penicillin G [89].

In the case of* B. cereus*, the bacteria—due to *β*-lactamase production—are insensitive to penicillin-related antibiotics (merely 1% of strains are susceptible to penicillin) and show resistance to erythromycin and tetracycline, for example, carbapenem [61, 93, 94]. We suppose that, due to problems with antibiotic treatments and improvement in bacterial drug resistance, these strains may be used as potential biowarfare agents. Therefore, for public safety, there must be known an agent to which these bacteria are susceptible.

Treatment with antibiotics beginning 1 day after the exposure to an aerosol with anthrax spores can protect against death. However, optimal protection isprovided bycombining antibiotics with vaccination. Vaccination is the best form of mass protection. The first anthrax animal vaccine was developed by Pasteur in 1881. Pasteur attenuated* B. anthracis* strains and proved that these strains could protect sheep from fully virulent strains [95]. Human vaccines emerged in the middle of the 20th century [9]. Human anthrax vaccine (anthrax vaccine adsorbed, AVA), currently licensed for use in the United States and the United Kingdom, consists primarily of protective antigen (PA) absorbed onto aluminum hydroxide [96, 97]. This vaccine was tested in guinea pigs, rabbits, and rhesus macaques by Fellows et al. [98].

According to FDA prescribing information concerning the observed side effects of AVA (BioThrax), local adverse reactions have been observed (especially at injection site), for example, tenderness, pain, erythema, edema, and arm motion limitation; (≥5%) as well as systemic adverse reactions: fatigue headache and muscle aches [99]. The currently available vaccines have a chemically complicated composition and it is believed that they are insufficiently purified [100].

AVA was originally prepared for individuals in high-risk occupations, like veterinarians, farmers, and laboratory personnel working with* B. anthracis* but was also used for military personnel [96]. About 150 000–200 000 American soldiers sent in 1991 to the war in the Persian Gulf were vaccinated against anthrax [101].

The use of appropriate animal models provides better understanding of the pathogenesis of human anthrax and the development of appropriate methods of prevention and treatment. Rabbits and nonhuman primates (NHPs), for example, rhesus macaques, are commonly used as animal models of inhalational anthrax. The pathological changes observed in rabbits and NHPs are similar to those observed in humans [102]. Savransky et al. showed that the pathology caused by the inhaled form of anthrax in guinea pigs is similar to that in both rabbits and NHPs, as well as in humans. Guinea pigs have alsobeen used in anthrax vaccine studies.

Another popular animal model used to test the sensitivity to virulent* B. anthracis* is the mouse. The mouse model is useful in studies on host resistance to anthrax and on pathogenesis, how the agent establishes infection in the host, and characteristics of the spore and vegetative bacilli. It is known that different mouse strains have various sensitivities to infection by both* B. anthracis* and anthrax toxin [103]. For instance, the BALB/c mouse strain is highly resistant, and strains such as A/J and DBA/2J are highly susceptible to infection [12, 99]. Interestingly, the susceptibility of mouse strains to lethal toxin (LT) does not necessarily correlate with its susceptibility to infection. For example, the susceptibility of A/J mice to anthrax toxin appeared to differ from the susceptibility to infection [99]. The rat and hamster, meanwhile, are important animal models for understanding the* B. anthracis* exotoxins, both LT and EF [12].

### 5.1. The Potential Use of Phage in Anthrax Treatment

The first phage therapy studies on* B. anthracis* were conducted by Cowles and Hale on mice [104]. The* B. anthracis* Thomas strain and bacteriophages which had been isolated from a malignant pustule, which were applied as therapeutics, were used in the experiment. The animals were inoculated (intraperitoneally) with 0.1 mL of bacteria (10^6^ cfu/mL) and 0.1 mL of bacteriophage (10^9^-10^10^ pfu/mL). The authors found that, only in the group inoculated with* B. anthracis* and bacteriophage mixture incubated 25 min before injection, 100% of mice survived. The results of this study also showed that only the phage, in high titer, quickly and permanently lysed the strain of anthrax used in the experiments.

Phages may be applied in phage therapy in the case of* B. anthracis* (also drug-resistant) infections [70]. For better effectiveness of therapy, phages active against* B. anthracis* should encode capsule depolymerases, to degrade the PDGA capsule that may be present in the bacterial surface. In this case phages may bind to the cell surface receptor of the bacteria and destroy these dangerous bacteria [54, 105].

Besides the whole phage particles, also endolysins can be applied in the therapy of anthrax. Endolysins are enzymes encoded in the bacteriophage genome and specifically lyse the peptidoglycan of the bacterial cell wall during the phage lytic cycle [106]. The enzymes may create new opportunities for the construction and production of genetically engineered enzymes for bacteria elimination, biocontrol, and experimental therapies. The endolysin PlyG isolated from the *γ* phage may be applied against* B. anthracis* (e.g., used as abiowarfare agent) [86]. Susceptibility of* B. anthracis* strains to *γ* phage infection and purified PlyG lysin isolated from this phage indicated that both of these agents have a narrow bacteriolytic spectrum—they especially showed high activity against almost only* B. anthracis* strains [86]. The authors decided to use isolates of streptomycin-resistant* B. cereus* RSVF1 strain because of the similarity of this strain to the* B. anthracis*. Lytic activity of lysin against this strain was the same as in the case of* B. anthracis* strains. In the study of Schuch et al., (2002) BALB/c mice were intraperitoneally infected with* B. cereus* RSVF1 (1.0 × 10^6^ cfu/mL) and, 15 min later, treated with 50 and 150 U PlyG. The application of lysin significantly rescued mice in comparison to untreated animals. Moreover, resistance to PlyG was not observed* in vitro* in either RSVF1 or EMS RSVF1 mutagenized strains (mutagenesis with the use of ethyl methanesulfonate). However, both Novobiocin (3.5 *μ*g/mL^−1^) and streptomycin (150 *μ*g/mL^−1^) resulted in bacterial resistance to these antibiotics. What is more is that the authors have demonstrated that RSVF1 strain that became resistant to the phage remains sensitive to PlyG. In bacterial culture, application of lysin caused morphological changes of bacterial cells and ultimately led to cell lysis. Also, purified lysin encoded by the Tsamsa phage is suggested to be used in* B. anthracis* biocontrol due to its broad lytic spectrum that lysed more strains belonging to the* B. cereus* group than complete phage and which goes outside* B. anthracis* strains [56]. Inal suggested that in the case of anthrax infections lysin should be applied as soon as possible, before the lethal level of toxin is reached [32].

Porter et al. described PlyB lysin which showed lytic activity against a* B. anthracis*-like strain (ATCC 4342). The enzyme has muramidase activity, whereas PlyG is an amidase [107]. It is presumed that this lysin may be a new defensive tool in the face of bioterrorism danger. Lysins have some advantages over phages as the capsule is not an obstacle for PlyG to access the bacterial cell wall and may destroy encapsulated forms of bacilli. They show high specificity, not disturbing another bacterial species, and strong enzymatic activity; moreover the enzymes allow destruction of bacteria within seconds or minutes [108]. In* in vivo* experiments it was showed that PlyG applied in mice intraperitoneally did not cause evident toxic effects [86]. Another prevalence of these enzymes is that the resistance to them is induced rarely or not at all in comparison with whole phage particles. What is more is that it was observed that purified lysin isolated from Tsamsa phage was characterized by broader lytic activity than it was observed in the case of phage host range [56]. This phenomenon may be useful for biocontrol and decontamination not only in the case of* B. anthracis* threat but also in the instances of other* B. cereus* group bacteria contamination.

Sozhamannan et al. suggested that applying a combination of two different phages (*γ* and AP50c) with different lytic spectra may be a better alternative for therapy of anthrax, phage-based diagnostics, and disinfection of areas contaminated with anthrax bacilli [66]. Similarly, Inal stated that a phage cocktail (which has the ability to lyse most* B. anthracis* strains) should be prepared and tested as an optimal antianthrax agent [32]. Also, Porter et al. proposed feasible application of the combination of two different lysins, PlyB and PlyG, which exhibit different lytic activity and cleave different peptidoglycan bonds [107].

It was suggested that phages, especially a combination of different phages, may be used in a spray form applied to skin and clothes surface and into the respiratory tract [86].

### 5.2. *B. anthracis* Spore Decontamination

Although the use of phagesagainst* B. anthracis* is mainly limited to vegetative forms of the bacteria, there are phages that may be used for removing anthrax spores. Anthrax spores are not metabolically active, and they may be inactivated by physical methods (gamma irradiation, ultraviolet light, and high pressure) that are not safe for humans [71, 109]. There is a need to find a method of disinfection that is highly effective and safe. This form of* B. anthracis* is the most dangerous one as a potential terrorist bioweapon.

The spore cortex is protected by a proteinous coat against, for example, lysozyme. In the germinating state the porosity of coat is increased (even during 10 minutes of incubation in conditions inducing germination) [86]. Fu et al., usingcryoelectron tomography, describedthe structure of the SBP8a phage active against both vegetative and spore forms of* B. anthracis* and the molecular mechanism of phage infection [110]. The phage showed the possibility to recognize and adhere to the surface of spores and eject its DNA inside the spore by the conformational changes of phage structures (at high SBP8a concentration, approximately 10^8^ pfu/mL).

Application of phages (isolated from soil) in the aerosol form to germinated spores of the* B. anthracis* Sterne strain caused effective destruction of spores, but the effect was mainly observed when high titer lysate was applied (2.8 × 10^8^ pfu/mL, 3.5 × 10^8^ pfu/mL) [59]. The* B. anthracis* Sterne strain is a surrogate for virulent* B. anthracis*, which enables safe conduct of experiments on the avirulent* B. anthracis* Sterne strain and, according to data, the substitution does not significantly change or limit the results of the studies [71]. But using this strain guarantees safety—especially laboratory personnel who work on the* B. anthracis* are exposed to the risk of anthrax infection—and gives the possibility to conduct research on these dangerous bacteria.

As was observed, phages that are used against anthrax spores should be resistant to harsh environmental conditions, for example, dryness, ultraviolet radiation, extreme temperatures, and bodily fluids,to maintain ability to kill bacteria [71]. This feature would be important especially in the case of the disinfection application of spores (because of their high resistance to different factors).

## 6. Bacteriophages in Foodborne Pathogen Disinfection

There may exist the possibility to use other pathogens belonging to the* B. cereus* group in a bioterrorism attack. Bacteriophage typing may be useful for detecting food contamination with* B. cereus* [111], due to the fact that this method is cheap and convenient and seems to be fairly accurate [72]. The FDA approved the use of bacteriophages in order to guarantee food disinfection [112, 113]. There is a possibility and permission to apply bacteriophages providing food safety. To inhibit* B. cereus* contamination, the use of BCP78 phage isolated from fermented food was proposed [112].

Bacteriophages infecting* B. cereus* may be helpful in destroying this foodborne pathogen. For example, two phages, FWLBc1 and FWLBc2, which were isolated from soil, reduced the pathogen in mashed potato (by >6 log_10_ cfu/mL during 24 h). Because of the phages' narrow lytic activity, it has been suggested to use them as a component of phage cocktails [114]. This high specificity may constitute a disadvantage in using phages against foodborne pathogens, due to the complex composition of bacteria that contaminate food. A broad spectrum of inhibition of bacterial growth has been shown for Bc431v3 phage. It lysed bacteria belonging to the* B. cereus* group and* B. licheniformis*,* B. megaterium*, and* B. psychrosaccharolyticus*. The BPS10C and BPS13 phages that showed lytic activity against* B. cereus* were able to completely inhibit bacterial growth (bacteria belonging to the* B. cereus* group) for up to 6 h [115]. New phages with proven activity against* B. cereus* are still being isolated [116]. But it is extremely important that in their genomes phages do not encode genes responsible for lysogeny, toxin production, and genes affecting the pathogenicity of bacteria and antibiotic resistance [72]. The lack of them makes phage application safe for humans and increases phage application as a strategy of biocontrol of bacteria belonging to the* B. cereus* group.

Endolysins may be successfully applied in the case of* B. cereus* contamination [108]. For example, the LysB4 lysin isolated from the B4 bacteriophage was reported as the first endopeptidase among endolysins obtained from the* B. cereus* phages. Interestingly, the enzyme not only shows broad lytic activity against* B. cereus* strains but also lyses Gram-negative strains, for example,* E. coli* strains, in comparison with the phage lytic spectrum, which, most frequently, is limited to one* B. cereus* strain. This feature enables the enzyme to be an effective antibacterial agent active especially against foodborne pathogens. Furthermore, the lysin destroyed bacteria in merely 15 min and, according to Lee et al., this enzyme seems to be a perfect candidate as a biocontrol agent in the case of* B. cereus* contamination [61]. Endolysin BPS13 isolated from the BPS13 phage was highly temperature-stable; for example, it displayed lytic activity even at 100°C (suspended in glycerol) [115]. Yuan et al. isolated PlyBtSC33 endolysin from the* B. thuringiensis* BtSC33 phage [117]. The authors showed that this agent may be potentially used for disinfection purposes, had high temperature resistance, and showed a broad lytic spectrum (low lytic activity against* B. thuringiensis* but higher activity against* B. anthracis* and* B. cereus* strains). High thermostability may be useful in lysin application against food poisoning caused by* B. cereus*, especially in the heat treatment process. This endolysin may also be considered in anthrax treatment.

The lytic protein E33L that caused the lysis of* B. anthracis* was isolated from the genomeof* B. cereus* [118]. It was an N-acetylmuramoyl-L-alanine amidase, active against both* B. anthracis* and closely related strains belonging to the* B. cereus* group. This enzyme induced complete lysis already in nanomolar concentrations* in vitro* (almost 99% lysis of* B. anthracis* was achieved at 50 nM in 60 min). What is more is that the protein was active against* B. cereus *strains. An advantage of this agent is that the enzyme does not seem to be degradable by bacterial proteases, and furthermore it showed significantly higher lytic activity than* Bacillus*-phage-encoded endolysins.

The biothreat danger is a real possibility, and regardless of how the attack occurs (through water, air, mail, food contamination, soil, insects, and public transport) people should have a foolproof tool for rapid detection and identification and a possibility to treat patients from these dangerous (probably drug-resistant) pathogens. The control of* B. cereus* group bacteria, especially* B. anthracis*, is important in prevention and detection of bioterrorist attacks involving food contamination with regard to human health safety and economic reasons. We suggest that phages (whole particles or their purified endolysins) may constitute a good prospect in this area.

## 7. Concluding Remarks

The past two decades have proved that bioterrorism is a real threat which needs to be properly controlled. Recent developments in phage therapy confirm that it may provide a reliable countermeasure preventing serious consequences of a terrorist attack using deadly bacteria, especially those resistant to antibiotics. Phage-mediated elimination of* Bacillus cereus* group bacteria, especially* B. anthracis*, seems to be an efficient tool against the potential use of such bacteria as a terrorist bioweapon.

## Figures and Tables

**Table 1:** *Bacillus anthracis* phages and their characteristics.

Name of phage	Type of life cycle	Short description	Phage host specificity	Possible application
W*β*	Lysogenic	Belongs to *Siphoviridae.* Inability to infect encapsulated cells [4].	Infects all 171 tested nonencapsulated strains [50], but does not infect other *Bacillus* strains.	Preparing bioluminescent reporter bacteriophage for *B. anthracis * **detection **in clinically relevant samples [56] and providing an antibiotic susceptibility profile [4].

Gamma phage (*γ*)	Lytic	Belongs to *Siphoviridae* [57]. Cannot bind to GamR receptor on bacterial surface and does not encode a PDGA depolymerase. Encodes a fosfomycin resistance gene [58].	*B. anthracis* 1584; 211; SL 1809; Sterne 34F2 [51]. Not active against *B. anthracis* Ames strain that produces capsule. Strains that do not encode the pX01 plasmid are more susceptible to phage *γ* than strains that possess the plasmid [62].	**Identification** of *B. anthraci*s strains and its differentiation from other similar strains from *B. cereus *group.

AP50	Lytic	Belongs to *Tectiviridae *[55], isolated from soil. Infects only *B. anthracis* strains. Does not lyse strains belonging to different *Bacillus *spp. The lysogenic mutant AP50c is characterized by very high killing efficiency [63].	Narrow host range [64]. Lyses 33% of *B. anthracis* strains [1]. This phage may infect bacterial strains that are resistant to *γ* phage [63]. It does not infect the *B. cereus* ATCC4342 strain, which infects the *γ* phage.	Probable use **in therapy** of anthrax. It is suggested to be used in **typing and biocontrol** of *B. anthracis* [65].

Fah	Lytic	Belongs to *Siphoviridae *[66].	*B. anthracis* 1584; 211; SL 1809; Sterne 34F2 [54]. Narrower lytic spectrum. Lyses 73–89% of *B. anthracis* strains [1, 66].	Probable use **in therapy** of anthrax.

Worm intestinal phage 1 (Wip1)	Lytic	Belongs to *Tectiviridae *[67]. It was isolated from the intestinal tract of *Eisenia fetida* worms. [52].	Exhibits a narrow host range highly specific to *B. anthracis* [67]. Does not infect the *B. cereus* ATCC4342 strain, which infectsthe*γ* phage [52].	Potentially useful diagnostic tool for efficient **identification** of *B. anthracis*; may be labelled and applied in organism for rapid readout [61].

Giraffe phage	?	Belongs to *Siphoviridae *isolated from giraffe faeces in a zoo (Long Island) [68]. This phage shows a rapid lysis phenotype.	Lyses the ciprofloxacin-resistant *B. anthracis* strain HS2-7 [68].	Possible use **in therapy **when infection is caused by antibiotic-resistant *B. anthracis *strain [67].

F7	Lytic	Isolated from bovine faeces. Belongs to *Siphoviridae *[51].	*B. anthracis* 1584; 211; SL 1809; Sterne 34F2; *B. cereus *ATCC13472; *B. cereus *ATCC* 10876; B. thuringiensis *ATCC 33679 [51].	Probable use **in therapy** of anthrax.

F9	Lytic	Isolated from bovine faeces. Belongs to *Siphoviridae* [51].	*B. anthracis* 1584; 211; SL 1809; Sterne 34F2; *B. cereus *ATCC13472; *B. cereus *ATCC* 10876; B. thuringiensis *ATCC 33679 [51].	Probable use **in therapy** of anthrax.

vB_BanS-Tsamsa	Lysogenic	Isolated from carcasses in Etosha National Park in Namibia. Belongs to *Siphoviridae. *Has the largest sequenced genomes of *Bacillus* siphovirus.Purified endolysin encoded in genome of this phage has broader spectrum than the phage. The largest siphovirus known to infect *Bacillus* strains [54].	Infects also strains belonging to *B. cereus* and *B. thuringiensis* [54]. Did not lyse the *B. anthracis* PAK-1 strain (resistant to both *γ* and cherry phage). Moderate specificity to *B. anthracis*.	Use of purified phage endolysin in *B. anthracis * ** biocontrol.**
